# Management and prevention of in-hospital cardiac arrest: present and future

**DOI:** 10.1038/s44325-024-00009-7

**Published:** 2024-07-02

**Authors:** Jonathan Vo, Faye L. Norby, Paul Marano, Yuri Matusov, Kyndaron Reinier, Joseph Ebinger, Henry Halperin, Sumeet S. Chugh

**Affiliations:** 1 Center for Cardiac Arrest Prevention, Department of Cardiology, Smidt Heart Institute. Cedars-Sinai Health System, Los Angeles, CA USA; 2 Division of Epidemiology and Community Health, University of Minnesota School of Public Health, Minneapolis, MN USA; 3 Department of Medicine, Division of Pulmonary and Critical Care Medicine, Cedars-Sinai Medical Center, Los Angeles, CA USA; 4 Division of Cardiology, Heart and Vascular Institute, The Johns Hopkins University, Baltimore, MD USA

**Keywords:** Cardiovascular diseases, Ventricular fibrillation, Ventricular tachycardia

## Abstract

Cardiac arrest is most commonly defined as the cessation of cardiac mechanical activity requiring either delivery of chest compressions and/or defibrillation. The condition is often subdivided into in-hospital cardiac arrest (IHCA) and out-of-hospital cardiac arrest (OHCA) based on different locations, but also differences in epidemiology, natural history, co-morbidities, process of care, and provider characteristics. Both are complex conditions that warrant ongoing research to improve management, but IHCA appears to have received disproportionately less investigative attention. Recent reviews of over 150 randomized controlled trials (RCTs) conducted between 1995 and 2019 reported that the vast majority (>80%) were focused on OHCA, approximately 10% on both and <10% were focused solely on IHCA. In this review, we will provide an overview of current knowledge regarding IHCA epidemiology, management and prevention, while also identifying opportunities for future research.

## Incidence and outcomes

There are relatively few registries and data archives for IHCA as compared to OHCA. One of the largest is the American Heart Association (AHA) Get With The Guidelines-Registry (GWTG-R)^1^. The GWTG-R is a prospective, multicenter, voluntary registry that is sponsored by the AHA. Based on the GWTG-R, there were approximately 292,000 adult IHCAs occurred in the US (9.7 IHCA per 1000 admissions) in 2017, which represents an increase compared to 2008^1,2^. Based on GWTG-R data, the majority (~80%) of IHCA presenting rhythms are non-shockable (pulseless electrical activity (PEA) or asystole)^3^. Although the GWTG-R is one of the largest in the US, participation in the registry is voluntary, and only a minority of hospitals in the US are included. Therefore IHCA incidence may be underestimated by this registry. Additionally, the GWTG-R limits inclusion to cardiac arrests where resuscitation was attempted, and there was a hospital or unit-wide resuscitation emergency response, also contributing to potential underestimation of IHCA^1,2^. Despite these limitations, the GWTG registry is widely used given the prospective, multicenter design of the registry.

The IHCA registries generally measure survival outcomes up to one year following hospital discharge. There is a lack of studies reporting longer follow-up likely due to the challenges related to continued communication with survivors. Despite the rising incidence of IHCA events in the US, both survival to discharge and survival to one year after IHCA appear to be improving. An analysis of the GWTG-R database showed improved survival to hospital discharge following IHCA between 2000 and 2009 (13.7% to 22.3%)^4^. One-year survival following IHCA also increased between 2000 and 2011 (8.9% to 15.2%)^3^. A separate meta-analysis of 40 studies similarly showed an improvement of 1-year survival after IHCA during 1985-2018, though 1-year survival after IHCA continues to remain poor at 13.4%^5^. These improvements in survival are seen for both shockable rhythms (ventricular fibrillation(VF)/pulseless ventricular tachycardia (VT)) as well as non-shockable rhythms (pulseless electrical activity (PEA)/asystole)^3,4^. A 2021 study examining data from the US National Inpatient Sample (NIS) Data showed an improvement in survival in PEA/asystole presenting rhythms from 18.9 to 30.2% and an increase in VT/VF presenting rhythms from 29.8 to 39.7% between 1998 to 2018^6^. While the US NIS database is a nationally representative large inpatient registry, the reliance on ICD codes for determination of IHCA leads to potential for inaccuracy and variability due to coding errors. Survival rates for non-shockable rhythms have increased more relative to shockable rhythms; however, non-shockable rhythms continue to have a lower survival rate as compared to shockable rhythms, and an initial presenting rhythm of VF/VT (as opposed to PEA/asystole) has been shown to be a positive predictor of survival and neurologic outcome^3,6,7^.

**Fig. 1:**
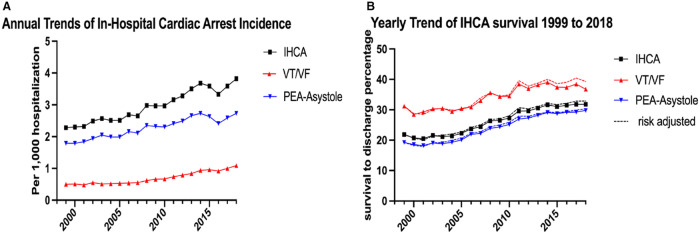
IHCA incidence and outcomes. Trends in incidence of IHCA and presenting rhythms (Panel **A**) and IHCA survival outcomes (Panel **B**) in the US ^6^. Reprinted with permission.

The significantly lower survival rates for IHCA presenting with non-shockable rhythms when compared to shockable rhythms are due to two important factors. Firstly, shockable rhythms can be treated by defibrillation, but there are no specific treatments for non-shockable rhythms. Second, individuals presenting with non-shockable rhythms tend to be older and have a higher burden of co-morbid conditions. A recent study from the Danish in-hospital cardiac arrest registry (DANARREST) reported that higher age, female sex, and several comorbidities including obesity, renal disease, and lung cancer were associated with an initial non-shockable rhythm^8^. Several studies have evaluated differences in shockable versus non-shockable rhythms with the goal of improving mechanistic understanding and developing new treatments for non-shockable rhythms^9–13^. These have largely been conducted in the out-of-hospital cardiac arrest setting. This is another area where more studies need to be performed in the IHCA setting. Improvements in overall survival over time for IHCA as well as improvements stratified by initial rhythm are shown in Fig. 1^6^.

One notable exception to this steady increase in survival rates is during 2020 with the onset of the COVID-19 pandemic in which rates of survival to discharge for IHCA decreased among patients regardless of COVID-19 infection^14^.

## Etiology of IHCA

Causes of IHCA have been categorized by the H’s and T’s: hypoxia, hypovolemia, hydrogen ion excess, hypo/hyperkalemia, hypothermia, tamponade, toxins, tension pneumothorax, and thrombosis. However, recent studies have shown that a sizeable proportion of IHCAs may result from etiologies not accounted for by the H’s and T’s^7,15^. A systematic review and meta-analysis from 2022 showed that the most frequent etiologies of IHCA were hypoxia, acute coronary syndrome, arrhythmias, sepsis, heart failure, and hypovolemia, while other conditions among the traditional H’s and T’s accounted for just 0.10 to 3% of cardiac arrest^16^. These findings have important implications for management of IHCA. Identification of the likely IHCA etiology allows for directed treatment of the underlying disease condition, an approach that improves survival to hospital discharge by 19%^15^. Recent studies report that cardiac etiologies may account for approximately half of all IHCA with the remaining half having non-cardiogenic causes^17,18^. Also, a few relatively small studies suggest that the proportion of IHCA with cardiac causes may also be increasing^19,20^, but this finding needs to be evaluated in larger and more diverse populations. Additionally, it should also be noted that a limitation of several published studies is the challenge of determining if the IHCA is due to a primary cardiac condition or secondary to a non-cardiac condition, for example, hypoxia due a lung condition. This phenomenon introduces bias in the data and should be taken into consideration when interpreting findings of IHCA investigations.

## Effects of sex and race on IHCA outcomes

The role of sex in neurological outcomes and survival from IHCA continues to be debated. Earlier studies reported that women have a nearly two-fold higher mortality rate following IHCA as well as a trend towards worse neurologic outcomes when compared to men^21,22^. However, a subsequent 2023 retrospective study using data from the Swedish Cardiopulmonary Resuscitation Registry (SCRR), the first such registry ever established, concluded that women actually have better survival rates compared to men after controlling for a comprehensive set of variables including socioeconomic status, race, and co-morbidity^23^. Notably, prior studies have reported that women with IHCA are older and more likely to present with non-shockable rhythms as compared to men, and both of these factors are negative predictors of ICHA survival. This may be one possible explanation for the divergent findings between the earlier studies and the 2023 study, but further investigation is needed^22^. Importantly, the SCRR only includes patients for whom resuscitation is attempted, limiting the generalizability of these findings to overall IHCA.

Data regarding the effects of race on survival is more consistent, with multiple studies demonstrating that Black patients were less likely to experience ROSC or survival to discharge as compared to White patients^24–26^. Black patients were also more likely to have at least moderate neurological disability at discharge^24^. These disparities remain even after adjusting for higher rates of renal insufficiency, respiratory insufficiency, and sepsis as well as lower rates of prior MI in Black patients^24^; however, given that this analysis was done using GWTG-R data, similar limitations as above apply to this result. Among IHCA survivors that were discharged from the hospital, Black patients also had lower 1-,3-,and 5-year survival compared to White patients^24^.

These racial disparities have been attributed to several possible factors. A 2009 study of Black patients experiencing shockable IHCA demonstrated that Black patients were more likely to receive treatment at hospitals with worse outcomes^26^. On the contrary, among Black patients with IHCA, due to non-shockable rhythms, hospital quality and patient characteristics were not significantly related to race-specific outcomes^25^. Unmeasured characteristics such as pre-existing do not resuscitate orders^25^, and post-discharge care disparities have been postulated^24^; however, more work is needed to explain these disparate outcomes.

**Fig. 2:**
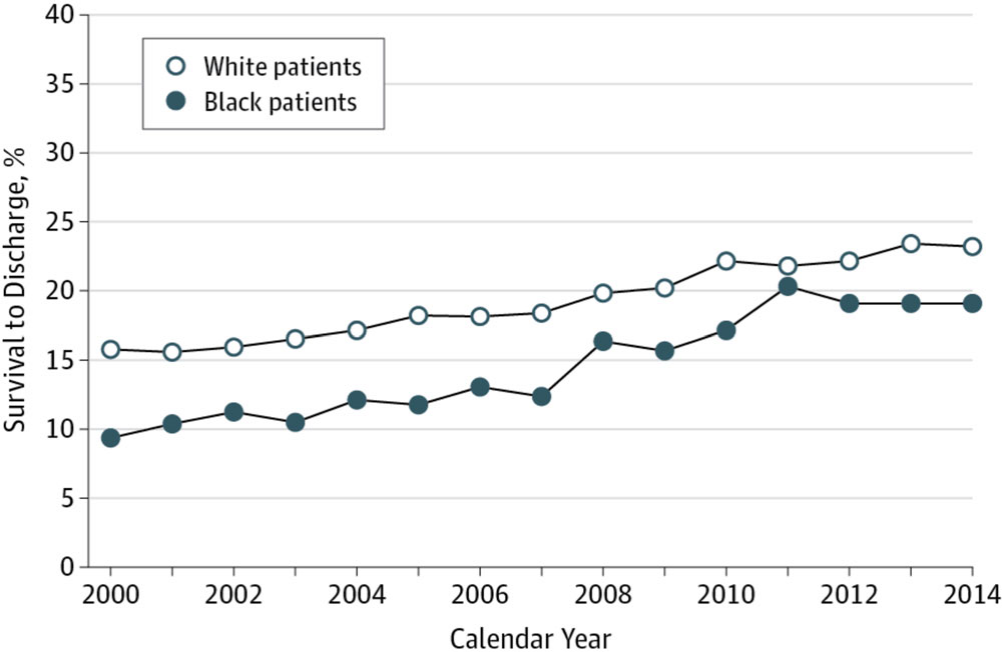
Decline of racial differences in survival to hospital discharge over time in the United States^27^. Reprinted with permission.

Although Black patients have a lower survival rate compared to White patients, this racial gap has narrowed over the past decade. Based on the GWTG cohort from 2000 to 2014, risk-adjusted survival for Black patients improved from 11.3% in 2000 to 21.4% in 2014, while white patients’ survival improved from 15.8% in 2000 to 23.2% in 2014 as seen in Fig. 2^27^. Notably, Black patients experienced a larger increase in survival-to-discharge as compared to White patients. Improvements in survival in both groups were partially mediated by improvements in resuscitation quality as determined by confirmation of endotracheal tube (ETT) placement, time to first chest compression of less than 1 minute, time to first defibrillation of less than 2 minutes, and administration of epinephrine within 5 minutes^27^. The larger improvements in survival observed in Black patients compared to White patients were attributed to improved ROSC in Blacks. Hospitals with higher proportions of Black patients initially had lower initial survival rates but experienced the largest increases in survival over time^27^. Significantly more work is needed to understand how race and ethnicity affect IHCA outcomes.

## Regional variation in outcomes of IHCA

**Fig. 3:**
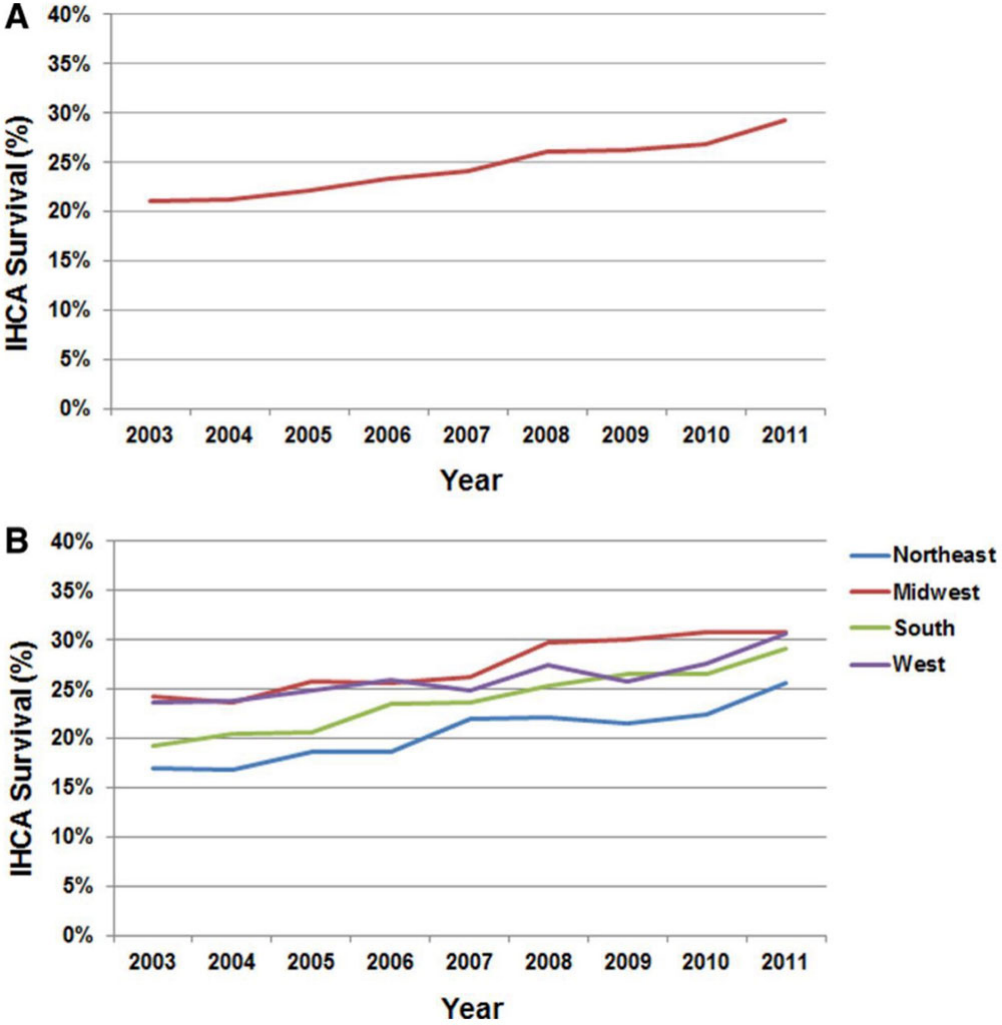
Regional Differences in survival. Panel **A** shows IHCA survival to discharge rates in the US as a whole, and Panel **B** shows IHCA survival rates subdivided by geographic region^29^. Reprinted with permission.

Although overall IHCA incidence is rising and survival outcomes appear to be improving within the US, there exists a large inter-hospital variation in survival to discharge. In a 2014 cohort study using the GWTG-R data, adjusting for patient factors such as age, sex, and comorbidities, top decile academic hospitals had a 22.8% median adjusted survival rate while bottom decile academic hospitals had an 11.9% median survival rate^28^. This inter-hospital variation held true for non-academic hospitals with top decile non-academic hospitals demonstrating a 22.5% median adjusted survival rate while bottom decile hospitals had a 12.9% median survival rate^28^. There was no significant difference in survival rates between top decile academic hospitals as compared to top decile non-academic hospitals^28^. A 2015 study examined United States geographic and individual state variations in IHCA incidence and survival, noting that IHCA incidence is highest in US western states (3.73 per 1000 admissions) and survival to discharge significantly higher in the midwest (27.7%), west (26.2%), and south (20.7%), even after adjusting for demographics, comorbidities, and initial cardiac arrest rhythm^29^. Differences in IHCA survival between individual states were also noted with highest risk-adjusted survival being in Wyoming at 40.2% and the lowest in New York at 20.4%^29^. Survival to discharge also appears to be increasing in each of the four geographic regions, as shown in Fig. 3^29^. Although there were large variations in incidence of IHCA regionally and between individual states, the prevalence of comorbidities was not significantly different^29^. Patient demographics as well as size and location of the hospital may play larger roles in these regional outcome variations. IHCA incident rates were higher in urban hospitals and hospitals with higher proportions of Black patients, and lower in larger hospitals^30^.

## Effects of IHCA location within the hospital

The location of the IHCA event within the hospital seems to affect the incidence and survival of IHCA. Three studies have demonstrated that although IHCA incidence was higher in ICU settings, survival to discharge was lowest in unmonitored bed settings and highest in either the ICU or a monitored general medicine ward bed^31–33^. Specifically, one study showed an unadjusted mean event rate of 0.337 events per 1000 patient-bed days in the ICU, 0.109 events per 1000 patient-bed days in the monitored ward, and 0.134 events per 1000 patient bed-days in the unmonitored ward, but adjusted survival rates of 19.3% in the monitored ward setting, followed by 14.0% in the ICU, and finally followed by 10.6% in the unmonitored ICU setting^32^. Another study showed patients with monitored and/or witnessed IHCA events were over twice as likely to survive to hospital discharge, and, furthermore, monitored patients were more likely to have favorable neurologic outcomes^33^. The higher incidence in ICU settings likely reflects the acuity and higher disease burden and co-morbidities in ICU settings, but the higher survival rates in ICU and monitored wards are potentially the result of increased monitoring, higher nurse-to-patient ratios, and better availability of crash carts and other resources for advanced cardiac life support.

## Prevention of IHCA

Based on the setting, IHCA prevention can divided into two complementary approaches, hospital-based and pre-hospital. Hospital-based approaches include early warning scores/systems and the more novel use of signal analysis and machine learning to predict imminent IHCA. This can lead to prompt in-hospital interventions following an improved and rapid response to patients at risk of imminent SCA. The pre-hospital approach currently consists of measures that increase the speed of EMS contact and initiation of medical therapy to prevent IHCA.

### Hospital-based prevention

Initial care of IHCA begins with monitoring and early recognition. This initial link in the chain of survival for IHCA differs significantly from OHCA which depends on bystander involvement followed by 9-1-1 call-based emergency medical response^34^. Hospital-based prevention of IHCA has been recently facilitated by the use of rapid response teams (RRTs) and proactive rounding in the hospital which reduce both IHCA incidence and associated mortality^35,36^. Proactive rounding mostly consists of the deployment of RRT nurses to identify high-risk patients for preemptive interventions. Other rapidly advancing efforts that target improved monitoring and early recognition of IHCA include early warning systems (EWS) and triage tools^37–39^. EWS has been in use since at least the early 2000s, and the National Early Warning System (NEWS) was first developed and deployed in the UK in 2012. This system consists of seven vital sign parameters (respiratory rate, oxygen saturation, body temperature, systolic blood pressure, heart rate, and level of consciousness), and each is scored from 0 to 3 depending on their deviation from normality. A score of at least 7 prompts an RRT or MET to assess the patient emergently. NEWS was reported to display a high discriminatory ability for prediction of deteriorating patients and mortality within 24 hours^37,40^. Several other EWS have subsequently been developed including the NEWS2 as well as other modified early warning systems, all of which typically include multiple vital signs parameters as well as laboratory values^38,41–43^. Although these EWS have been effective in the early detection of sick patients and patients with high risk of mortality within 24 h, they display an overall modest ability to specifically predict IHCA, and the effectiveness of these systems in decreasing IHCA incidence appears to be controversial^38,44,45^.

Advances in technology are being evaluated and deployed at a rapid pace to facilitate accurate prediction of IHCA. These include artificial intelligence (AI), signal/waveform analysis, and biometrics. One of the most promising of these is the deep learning-based early warning systems (DEWS). First described in 2018, DEWS is a deep-learning neural network that uses an input of 4 vital signs (heart rate, systolic blood pressure, respiratory rate, and body temperature) and measures the risk of cardiac arrest from these inputs^46^. In a subsequent retrospective study, DEWS showed superior IHCA predictive ability as well as a lower false alarm rate compared to the Modified Early Warning Score (MEWS; AUC 0.860 vs 0.754)^47^. Electrocardiographic waveform analysis is also a promising method for IHCA prediction. In a recent 2019 study, a deep-learning-based AI model that utilized 12-lead ECG inputs demonstrated an AUC of 0.913 for predicting cardiac arrest within 24 hours^48^. Another deep learning-based early warning system is the deep learning-based cardiac arrest risk management system (DeepCARS^TM^) which was first developed in in 2018 and approved by the Korean Ministry of Food and Drug Safety in 2021. This is another AI-based early warning system that again utilizes vital signs as inputs and a deep learning neural network to predict IHCA and has been shown in a prospective study to outperform NEWS and MEWS^49^. However, to maximize accuracy, these new approaches should also incorporate the comprehensive clinical profile of the patient, including de novo co-morbid events that occur during the index hospitalization. Such studies are still awaited.

### Pre-hospital approaches

These relatively new approaches are still being investigated but have the potential to improve IHCA outcomes in the future by decreasing time to treatment of underlying disease processes. One example is a digital application (Pulsara) that targets improvement in pre-hospital communication and EMS response times, potentially resulting in shorter care timelines for patients with suspected STEMI thereby decreasing myocardial damage and risk of subsequent IHCA^50^. Another is the STOP STEMI© medical application which has been shown to reduce door-to-balloon times by 22%^51^. Especially for IHCA associated with acute coronary events, longer-term primary preventative measures among the general population have the potential for significant benefit^52^. Additionally, these applications are mostly focused on coronary events and should be extended to other conditions commonly associated with IHCA.

## Early response and initial management of IHCA

**Fig. 4:**
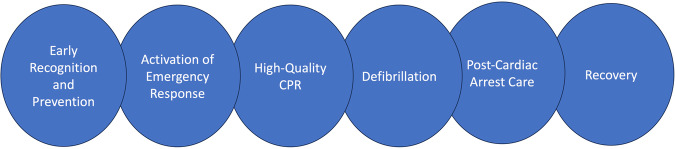
Chain of survival for IHCA^34^.

In addition to improved prevention measures, much of the improvement in IHCA survival outcomes can be attributed to better resuscitation and post-resuscitation techniques, widespread use of specialized code team responders, and adoption of guideline-directed therapy^6^. The AHA 2020 guidelines highlight the chain of survival for IHCA which begins with prevention, monitoring, and early recognition^34^ (Fig. 4). This has been more recently facilitated by the use of rapid response teams (RRTs) or medical emergency teams (METs) in the hospital which have been shown to reduce in-hospital mortality and cardiac arrest^35^.

The cornerstones of treatment for patients in cardiac arrest are chest compressions, defibrillation (if applicable), ventilation/oxygenation, and pharmacotherapy. Of these interventions, chest compressions and defibrillation have been a staple of management since 1960 and have shown to be the most critical part of CPR and should be initiated as soon as possible after cardiac arrest has been identified^53^. Delaying compressions by more than two minutes has been associated with decreased survival outcomes from 17.1% to 14.7%^54^. Defibrillation is another critical step in the treatment of IHCA with a shockable rhythm, and multiple studies have reaffirmed that delayed time (>2 minutes) to defibrillation is associated with lower survival rates, and this survival benefit extends out to 1-,3-, and 5-year survival^54–56^.

Optimal airway management continues to be explored and currently consists of bag-valve mask, endotracheal intubation, supraglottic airway device (SGA), and tracheostomy. Although intubation may allow for optimization of oxygenation and is a more secure airway compared to supraglottic airway devices, SGAs are faster/easier to place and require less training. Two RCTs that have compared intubation and SGA’s are the AIRWAYS-2 and PART trials. AIRWAYS-2 did not show a favorable functional outcome in the use of SGA’s as compared to endotracheal intubation, but PART did show improved 72-hour survival with usage of SGA’s^57,58^; however, both AIRWAYS-2 and PART were based on OHCA. There is the ongoing AIRWAYS-3 trial which compares intubation to SGA’s for IHCA^59^.

The recommendations for pharmacotherapy in IHCA management also continue to evolve. The usage of a combination vasopressin with epinephrine was originally recommended in the 2008 AHA guidelines on CPR, though this was later amended to sole usage of epinephrine based on a number of studies that showed no benefit with combination of both drugs^60–62^. There has been however more recent work on the combination of vasopressin-steroids-epinephrine that has been shown in three trials, with the most recent being the 2021 VAM-IHCA trial, to improve rates of ROSC, survival to hospital discharge, and favorable neurologic status^63–65^. Based on the results of these trials, the recent 2024 AHA guidelines for cardiac arrest have combination vasopressin-steroids-epinephrine as an alternative to epinephrine alone; however, currently no studies have found an advantage for combination therapy as compared to epinephrine alone^66^. The use of epinephrine itself, despite its inclusion in the VF/VT algorithm of IHCA, has only been shown to increase survival and ROSC outcomes for IHCA in patients with non-shockable rhythms alone, and current recommendations to use epinephrine for shockable rhythms are solely based on RCTs for OHCA^67,68^. To date, there are no RCTs for the use of epinephrine for IHCA; however, there have been multiple retrospective studies showing early epinephrine administration is associated with higher rates of ROSC, survival to discharge, neurologically intact survival, and 1 year survival^56,69,70^.

In addition to epinephrine, amiodarone and lidocaine are also guideline-recommended medications during resuscitation. Again, the use of these medications for IHCA is based on studies for OHCA rather than IHCA. Evidence for amiodarone is largely based on the ARREST trial which showed higher rates of survival to hospital admission in patients with OHCA due to shockable rhythms when treated with amiodarone^71^. The use of lidocaine is based on the ROC-ALPS (Resuscitation Outcomes Consortium-Amiodarone, Lidocaine or Placebo) study, which showed higher rates of ROSC and survival to hospital admission for lidocaine compared to discharge, though no difference compared to amiodarone^72^.

The use of dual sequential external defibrillation (DSED) is a novel form of defibrillation for refractory VF. DSED uses two shocks from two defibrillators in quick succession with the defibrillator pads placed in two different planes (anterior-posterior and anterior-lateral). Although this has been shown in prior case reports to lead to higher rates of termination of refractory VF, the recent DOSE-VF trial showed evidence of improved survival to hospital discharge as compared to patients who receive standard defibrillation^73,74^. Another novel tool that has become more widespread is the use of point-of-care ultrasound (POCUS) during cardiac arrest. Although prior studies have shown prolonged resuscitation pauses with POCUS, the Real Time Assessment with Sonography Outcomes Network (REASON) trial showed that POCUS has the ability to identify findings that respond to non-ACLS interventions as well as the significant result that 54% of patients classified as PEA had cardiac activity on ultrasound, highlighting the fact that finger pulse checks may not be sensitive enough^75,76^. One proposed method to minimize the resuscitation interruptions seen with POCUS is with the use of transesophageal echocardiogram (TEE). However, there currently remains a paucity of data and high-quality evidence for routine use of TEE in IHCA^77^.

### Post-resuscitation management of IHCA

#### Targeted temperature management

Current recommendations for post-resuscitation care hinge on hemodynamic support, adequate ventilation and oxygenation, targeted temperature management (TTM) to between 32 °C and 36 °C, and potential reversal of the underlying cause of cardiac arrest. There is no universally advised cutoff for maintenance of oxygenation or BP, though common recommendations include a 92-98% oxygen saturation level and a MAP > 65 or SBP > 90^53^.

Targeted temperature management has been an important strategy for improving neurologic outcomes after cardiac arrest, codified in resuscitation guidelines since two landmark randomized clinical trials in 2002 demonstrated improved neurologic outcomes and mortality with mild hypothermia compared with normothermia after OHCA with a shockable rhythm. These trials had several limitations, including small sample sizes, unblinded treating physicians, and a lack of standardization regarding withdrawal of care. While these trials only included OHCA survivors, the practice of targeted hypothermia was extended to patients with IHCA. Subsequent trials TTM1 and TTM2, in OHCA with predominantly (~75%) shockable rhythms, have addressed several of these limitations, and have demonstrated no benefit in terms of neurologic outcomes between hypothermia targeting 33 vs 36 °C (TTM1) and hypothermia targeting 33 °C vs targeted normothermia (TTM2)^78,79^.

Patients with IHCA and OHCA were both included in the HYPERION trial of patients with non-shockable rhythms, which demonstrated improved neurologic outcomes in patients with targeted hypothermia to 33 °C compared with normothermia^80^. However, this trial also had some limitations including how the primary outcome was assessed, unintended variation in body temperatures and the assumptions made regarding mortality. Most recently, there was a trial of TTM focusing specifically on patients with IHCA. The HACA-IHCA trial investigated the impact of mild hypothermia (32–34 °C) vs targeted normothermia in this population and was stopped early due to futility^81^.

In summary, more recent data suggests that normothermia and avoidance of fever may be sufficient in terms of temperature management in patients recovering from cardiac arrest. While the ACC/AHA 2020 guidelines were published prior to TTM2 and HACA/IHCA, the guidelines from the European Resuscitation Council and European Society of Intensive Care Medicine in 2022 now recommend continuous monitoring of core temperature and actively preventing fever > 37.7 °C for both OHCA and IHCA, and have found insufficient evidence to recommend for or against cooling to 32–36 °C^82^.

#### Neurologic outcomes and prognostication

**Fig. 5:**
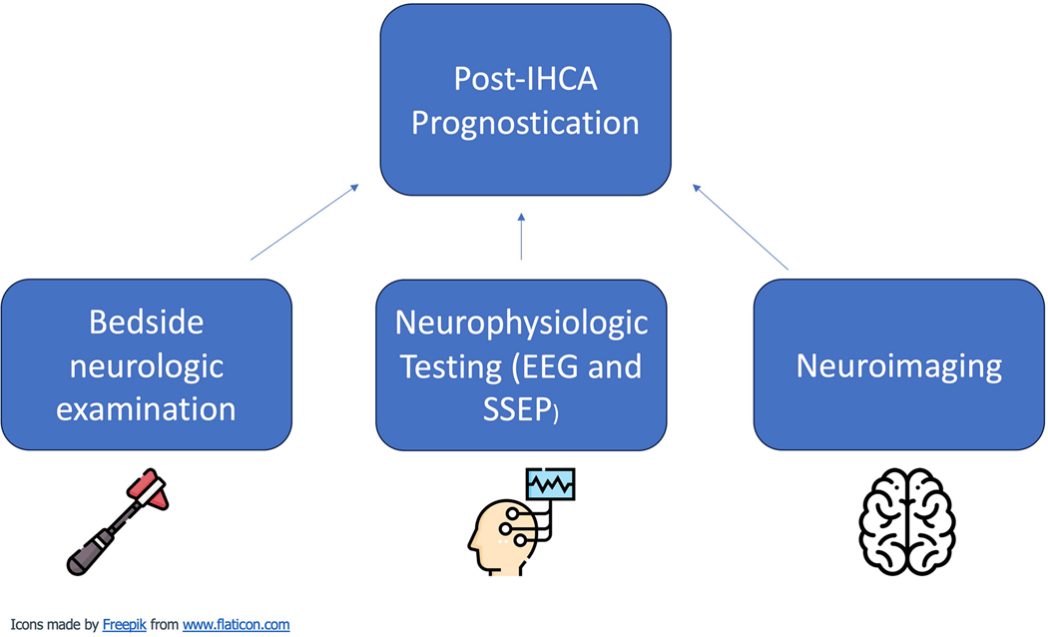
Elements of prognostication following IHCA.

Following IHCA resuscitation, neurologic outcome and prognostication comes to the forefront of management. Most deaths following ROSC after IHCA are from withdrawal of life-sustaining treatment due to poor neurologic prognosis^83^. Prognostication of neurologic recovery currently requires clinical assessment of neurologic function in combination with brain imaging, somatosensory evoked potentials (SSEP) and electroencephalography (EEG, Fig. 5).

It is important to recognize that these investigations must be conducted in the absence of other confounders such as metabolic derangements, abnormal vital signs, or certain medications^83^. Specific findings of value on neurologic examination include pupillary light and corneal reflex, gag and cough reflex, response to painful stimuli, and eye/facial movements^84^. In an attempt to improve the prognostication process, several prediction models have been developed. Of these, one of the most promising is the GO-FAR score which was originally developed in 2013 and comprises a number of pre-arrest predictor variables such as metastatic or hematologic cancer, altered mental status, impaired renal function, and diabetes mellitus^85^. GO-FAR was shown in a subsequent large 2023 systematic review and meta-analysis to be the only current prognostication model to have good performance while also being externally validated^86^. One of the most promising uses of prognostic scoring systems such as GO-FAR is to achieve the goals of care discussions. One of the fundamental medical ethical principles is informed consent, and these scoring systems allow practitioners the ability to convey a more accurate and realistic determination of neuro-prognosis. This can then be relayed to patients and families so that the appropriateness of CPR can be determined based on their goals and values.

## Conclusions

**Fig. 6:**
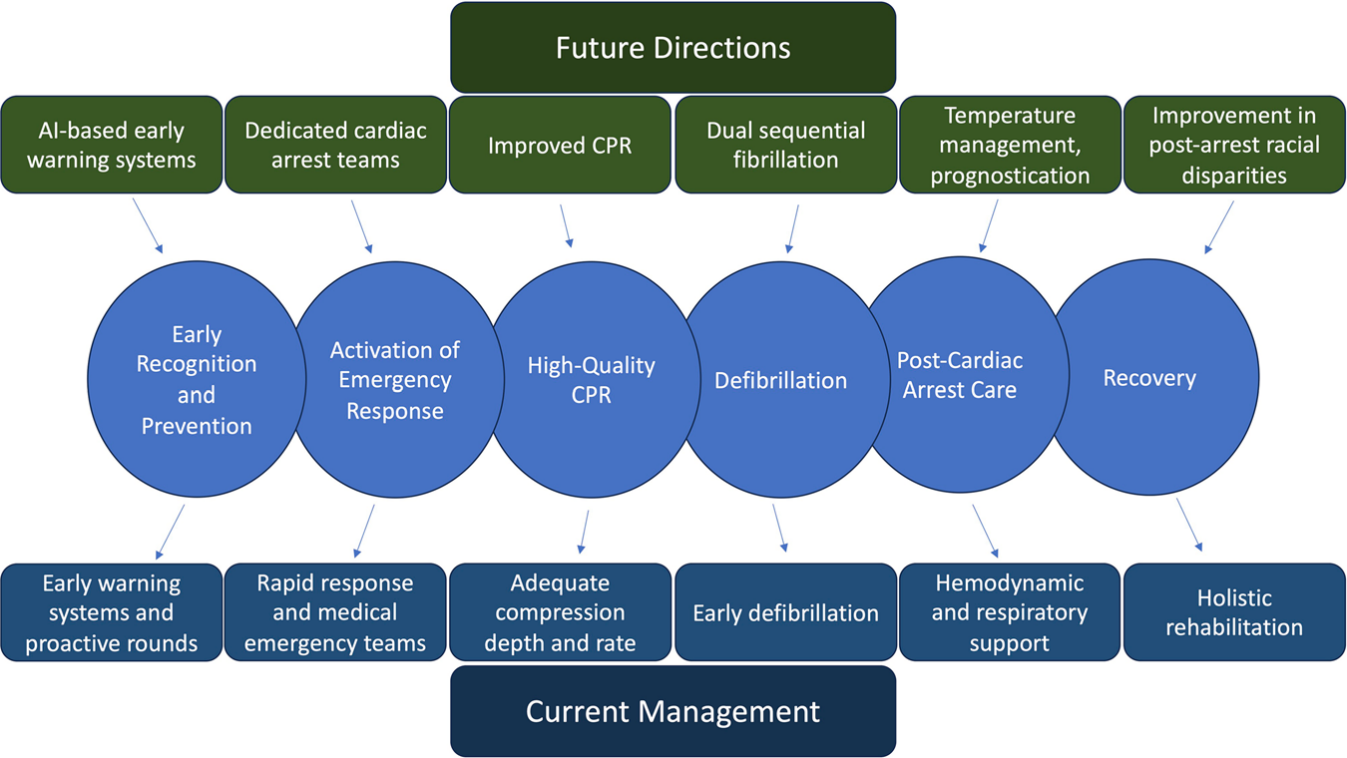
Schematic illustration of current IHCA management and the investigative opportunities that could enhance future management. Further developments in innovation and technology are needed for all aspects of the IHCA survival chain.

Despite advances in IHCA management and improvements in survival outcomes, IHCA continues to make a major impact on mortality. While we have discussed the present and future of the approach to IHCA in the United States context, conditions and approaches may vary based on specific regions at a global level. However, there is a high likelihood that the majority of successes, challenges and opportunities are shared at the global level. The last two decades have witnessed improvement in survival from IHCA in the US with the possible exception of the COVID pandemic. With the aging of the population as well as other factors, there is likely to be an ongoing evolution in etiologies of IHCA, a phenomenon that will, in part, contribute to a parallel evolution in IHCA approaches. Significant more work is needed before we can have a level playing field for IHCA management for both sexes as well as all races, ethnicities and socioeconomic groups. There is a need to standardize approaches so that regional disparities due to hospital size and location can be eliminated. A recent special report from ILCOR has laid out “ten steps toward improving IHCA quality of care and outcomes”^87^. To a large extent, much of this will be accomplished by the ongoing rapid developments in technology that will benefit both research and clinical implementation (Fig. 6). In particular, artificial intelligence tools such as deep learning may enable a quantum leap in refinement of currently existing early warning systems and prediction models to better monitor and prevent IHCA events^46,47,88^. Similarly, the ability to test novel technology and perform high-quality resuscitation trials more efficiently will benefit the cornerstones of IHCA management including CPR and defibrillation, temperature management and neuro-prognostication, all facilitating the ultimate goal of improving neurological outcomes as well as short and long-term survival from IHCA.
